# The Role of IL-6RA in UHMWPE Promotes Proliferation in Fibro-Like Synovial Cells

**DOI:** 10.1155/2018/3928915

**Published:** 2018-10-22

**Authors:** Xianlun Pang, Jiang Yang, Xiaoli Zhen, Hai Nie, Hao Qin, Lingyuan Huang, Lijie Zhang

**Affiliations:** ^1^Health Management Center, The Affiliated Hospital (TCM) of Southwest Medical University, Luzhou 646000, Sichuan, China; ^2^Department of Orthopedics, Yongchuan Hospital Affiliated to Chongqing Medical University, Chongqing 402160, China; ^3^Basic Medical College, Southwest Medical University, Luzhou City, Sichuan 646000, China; ^4^Department of Orthopedic Surgery, Sichuan Academy of Medical Sciences & Sichuan Provincial People's Hospital East Campus, Chengdu 610101, China; ^5^The Trauma Center of Military, Third Military Medical University, Chongqing 400038, China; ^6^Chengdu Lilai Biotechnology Co., Ltd., Chengdu 610101, China

## Abstract

UHMWPE granule could induce macrophages and inflammatory responses in interfacial tissues, which eliminated the wear debris of UHMWPE component and further induced dissolution of the surrounding bone, leading aseptic loosening. However, the mechanism of synovial cells, especially fibroblast-like synovial (FLS) cells response to UHMWPE, remains unknown. Herein we choose FLS cells as research object. Vimentin (+) CD68 (-) was identified by flow cytometry and immunofluorescent staining assay, and the cells were identified as FLS cells, which was consistent with the experimental requirements. The inhibitory evaluation showed that UHMWPE could significantly promote the proliferation and inhibit apoptosis of FLS cells in dose- and time-dependent manners and increase the levels of proinflammatory cytokines, including IL-6, IL-1*β*, TNF-*α*, PGE2, MMP2, and LOX. UHMWPE also can induce the expression of mIL-6R protein in FLS cells and further investigate the relationship between apoptosis and inflammation. Interestingly enough, when we added the interleukin-6 receptor antagonist (IL-6RA), the expression levels of proapoptosis-related proteins increased; in other words, UHMWPE-induced antiapoptosis diminished by IL-6RA (50 *μ*g/ml). Taken together, these findings clearly demonstrated that UHMWPE promote growth in FLS cells through upregulating inflammatory factors to produce antiapoptotic effect.

## 1. Introduction

The operation of joint replacement more than 1 million in the world each year, but the inefficient of joint 10 years, is nearly 10% [1]. Aseptic loosening is the major problem of arthroplasty longevity, which attributed to dissolution of the surrounding bone caused by the interaction between macrophages and wear particles [2]. The key of prevention is to restrain the osteolytic effect caused by excessive activation of local osteoclasts. Thus, mechanism research has great significance for develop the new drugs and methods to prevent artificial joint loosening.

Ultra high molecular weight polyethylene (UHMWPE) has been selected as materials of artificial joints more than 40 years due to its strong wear resistance and low friction coefficient, but it also has the strongest bone dissolving effect [3]. The initial step of periprosthetical osteolysis is macrophage swallowing the wear particles, further causing the release of cytokines and inducing inflammation in interface tissue [4]. In destructive arthritis, the most common cell types at the sites of invasion are resident cells of joint, in particular synovial fibroblasts [5]. When the artificial device with an UHMWPE component was implanted in joint with inflamed biologic environment, synovitis almost always exists, and the cells involved in the inflammatory response include mononuclear macrophages, fibroblasts, synoviocytes, and so on. They will produce cytokines (IL-1, IL-6, and TNF-a), proteolytic enzymes (metalloproteases), prostaglandins (PGE2), and destructive free radicals (H2O2, HO, and NO) to promote osteolysis and inhibit osteogenesis [6, 7]. Although there have been a study showing that the increase of free radicals on UHMWPE hip prostheses components may be due to inflamed synovial cell, which included degradation of UHMWPE components induced by synovial fibroblasts [8], the mechanism of the interaction between synovial fibroblasts and UHMWPE remains to be further studied.

Our aim in this study was to explore the interaction and mechanism between synovial fibroblasts and UHMWPE. We would clarify the role of UHMWPE in synovial cells from aspects in proliferation, apoptosis, and inflammation and further to explore the correlation between apoptosis and inflammation.

## 2. Materials and Methods

### 2.1. Reagents

UHMWPE (diameter: 55.62 ± 3.05 *μ*m) was purchased from Hoechst Aktiengesellschaft (Germany). The tocilizumab, a interleukin-6 receptor antagonist (IL-6RA) was obtained from Roche Pharma (Schweiz) Ltd. Additional reagents employed in the present study were commercially available and of analytical purity.

### 2.2. Tissues and Primary Synovial Cells

The synovial tissues from 8 cases and primary tissues from 8 cases were taken from RA patients in First Affiliated Hospital of Southwest Medical University (Sichuan, People's Republic of China) who needed joint replacements between July 2017 and September 2017. All samples were obtained with patient consent and with the approval of the Committee on Medical Ethics of First Affiliated Hospital of Southwest Medical University (Sichuan, People's Republic of China). After removing adipose tissue, the synovial tissues were cut into small pieces and then washed three times with 0.1% phosphate buffered saline supplemented with 1% penicillin-streptomycin (200 U/mL). Next, 2 mL of DMEM (Gibco, USA) without fetal bovine serum and 2 mL 0.4% type II collagenase (Gibco, USA) were added to the tissue pieces (each piece was 1×1×1mm, with a total of approximately ten pieces) and digested for 2 h at 37°C, 5% CO_2_. The nonadherent cells (the adherent cells were synovial tissue macrophage-like cells) were digested with 0.25% trypsin for 30 min at 37°C, 5% CO_2_. The mixture was filtrated through a 200-meshnylon net to remove the connective tissue, and the synovial cells were separated by centrifugation for 10 minutes at 8000 rpm. These subcultured cells were then cultured in DMEM at 37°C in an atmosphere of 5% CO_2_. The entire medium was supplemented with 10% heat-inactivated fetal calf serum (Gibco, CA, USA)) and 1% penicillin-streptomycin (100 U/mL). Cells were digested by 0.05% trypsin with 0.02% EDTA and subcultured.

### 2.3. Immunofluorescence Assay

After three periods of generation, the synovial cells covered approximately 60% of the slide. The cells were washed with phosphate buffered saline thrice and fixed in 4% paraformaldehyde for 15 min, and then cells were perforated by 0.2% Triton X-100 for 10 min at room temperature. After treatment, the cells were blocked by 5% BSA at room temperature for 30 min and incubated with primary antibodies at 4°C overnight. The primary antibodies included vimentin (Abcam, UK; ab92547) and CD68 (Abcam, UK; ab955) at 1:100 dilutions. Subsequently, cells were washed with PBST and incubated with secondary antibodies (Abcam, UK) at 1:2000 dilutions for 1 h at room temperature and finally stained with DAPI (Abcam, Cambridge, UK) for 5 min. The images of the cells were observed by a fluorescence microscope (Olympus Corporation, Japan) at a magnification of 400x.

### 2.4. Cell Viability Assay

UHMWPE was dissolved in dimethyl sulfoxide (DMSO; Sigma, USA) as a stock solution, stored at −20°C, and diluted with medium before each experiment. Cell viability was measured using the MTT assay. FLS cells were seeded at a density of 1×10^4^/well in a complete growth medium in 96-well plates. When the synovial cells covered approximately 60% of the plate, they were treated with different concentrations (0, 0.01, 0.1, and 1g/L) of UHMWPE for 24 h. MTT solution (5 mg/mL; Sigma, USA) was added to each single well of the 96-well plate (MTT plus fresh medium at a fixed ratio of 1:10, 100 uL/well) and incubated for 4 h. Then, remove supernatant and add DMSO (150 uL) to each well to dissolve the formazan crystal l produced from the MTT. Absorbance was measured at 490 nm on microplate reader (Thermo Fisher Scientific, USA). Absorbance can indirectly reflect cell vitality.

### 2.5. Edu Staining Assay

FLS cells were cultured in 12-well plates (3×10^4^/well) and incubated with 1.0 g/L UHMWPE for 7 d. Afterwards, the cells were stained with Edu (Invitrogen, USA) for 2 h at 37°C, 5% CO_2_. Then the cells were washed with phosphate buffered saline, incubated in 4% paraformaldehyde for 20 min, 2 g/L glycine for 5 min, and 0.5% TritonX-100 for 10 min, respectively. Finally, the cells were incubated in 1×Apollo buffer for 30 min at room temperature (20~25°C) in the dark, washed with 0.5% TritonX-100 and methanol thrice, respectively, stained with DAPI for 30 min at room temperature in the dark, and washed with phosphate buffered saline twice. The images of the cells were observed by a fluorescence microscope.

### 2.6. ELISA Assay

Samples of the cultured cell fluid were centrifuged at 5000 rpm for 10 min to obtain the supernatant. The supernatant was diluted three times. The expressions of interleukin 6 (IL-6), interleukin 1*β* (IL-1*β*), TNF-*α*, lipoxidase (LOX), prostaglandin (PGE2), and matrix metalloproteinase 2 (MMP2) in the supernatant dilution were detected with ELISA kit. Finally, the absorbance was measured at 450 nm on microplate reader (Thermo Fisher Scientific, USA)

### 2.7. Annexin V/PI Double-Staining and Flow Cytometry Assay

Cells were cultured in 6-well plates (1×10^5^/well) and treated with UHMWPE as described above. Annexin V-FITC-PI Apoptosis Detection Kit (BD Pharmingen, USA) was used to detect apoptosis according to the manufacturer's instructions. The cells were collected and resuspended in 100 *μ*L binding buffer (1×10^5^cells) with 5 *μ*L Annexin V-FITC and 5 *μ*L Propidium Iodide (PI). The cell suspension was incubated for 15 min at room temperature (20~25°C) in the dark and detected by FACSCalibur Flow Cytometer (BD Biosciences, USA) within 1 h. Generally, living cells were shown by Annexin V and PI double negative. Early apoptotic cells were indicated by Annexin V positive and PI negative (Q1-UR). Late apoptotic and necrotic cells were indicated by Annexin V and PI double positive (Q1-UR).

### 2.8. Western Blot Assay

The harvested cells were washed with phosphate buffered saline and lysed with RIPA lysis buffer (Boster, China), which was obtained total cellular protein. The protein concentration was tested by BCA Protein Assay kit (Boster, China). In order to detect the levels of protein expression, the protein samples were separated by 12% sodium dodecyl sulfate polyacrylamide gel electrophoresis (SDS-PAGE) and transferred onto a poly-vinylidene fluoride (PVDF) membrane through Bio-Rad II System. The membrane was blocked by 5% skim milk powder at room temperature for 1 h and then incubated with primary antibodies at 4°C overnight. The primary antibodies included caspase-3(Abcam, USA; ab4051), cleaved-caspase-3 (Cell Signaling Technology, USA; #9661), Bax (Abcam, USA; ab182733), Bcl-2 (Abcam, USA; ab692), IL-6R (Boster, China; A01425-1), *β*-actin (Bioss, China; bs-0061R), and GAPDH (Abcam, USA; ab8245) at 1:500 dilutions. Subsequently, membranes were washed with TBST and incubated with a 1:5000 dilution of HRP-conjugated secondary antibodies (Abcam, USA) for 1 h at room temperature. The protein bands were visualized by ChemiDocTMMP imaging system (Bio-Rad, USA). The GAPDH and *β*-actin were used as inner loading control, respectively. The gray value was analyzed by Image-ProPlus software.

### 2.9. Statistical Analysis

Data were presented as the mean ± standard deviation per group. Statistical analysis was made for multiple comparisons using analysis of variance and the Student's t-test. p-value < 0.05 was considered to be statistically significant.

## 3. Result

### 3.1. Identification of Human Primary Fibroblast-Like Synovial Cells

**Figure 1:**
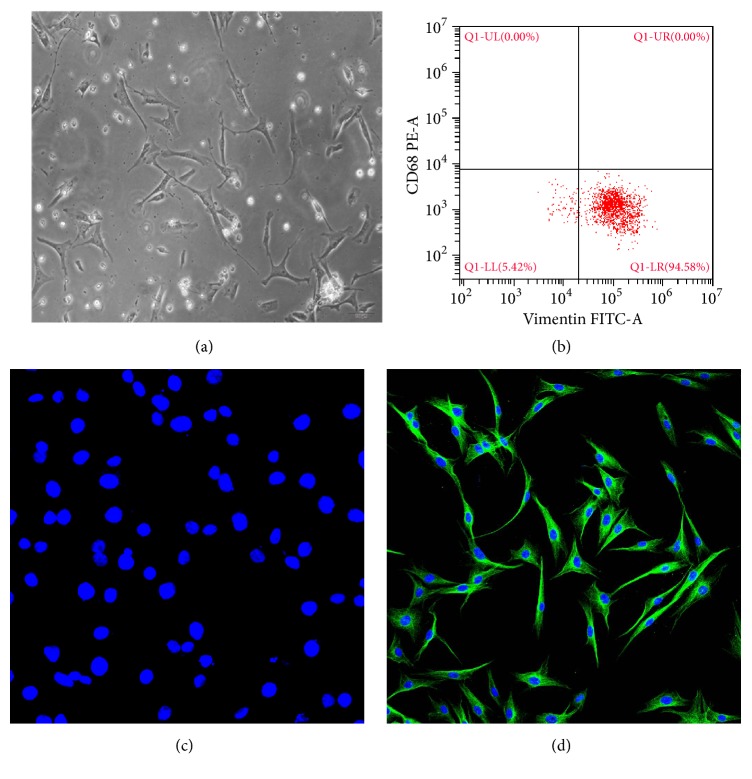
Culture and identification of fibroblast-like synovial cells. After primary separation and culture, the 5th generation cells were identified by flow cytometry and immunofluorescence. Morphological observation under light microscope (a). Identification of surface markers by flow cytometry (b). CD68 combined with DAPI staining (c). Vimentin combined with DAPI staining (d). The cells were observed on a fluorescence microscope (Olympus Corporation, Japan) at a magnification of 400x. Cell nuclei were visualized by DAPI staining in blue. The expression of vimentin was visualized by FITC staining in green. n=6.

After the 5th generation, most of human primary synovial cells were typical long fusiform, nucleus were in the center of the cell, and the shape was round or oval, with obvious nucleoli, showing a typical fibroblast-like cell morphology. From the cell surface marker detected by flow cytometry, results showed that the proportion of vimentin (+) CD68 (-) was 94.58%. Immunofluorescent staining verified the results of flow cytometry. As shown in Figures 1(c) and 1(d), cell nuclei were visualized by DAPI staining in blue; the protein of vimentin was visualized by FITC staining in green and mainly expressed in cytoplasm, while there was no expression for the protein of CD68.

### 3.2. The Effects of UHMWPE on Viability of FLS Cells

**Figure 2:**
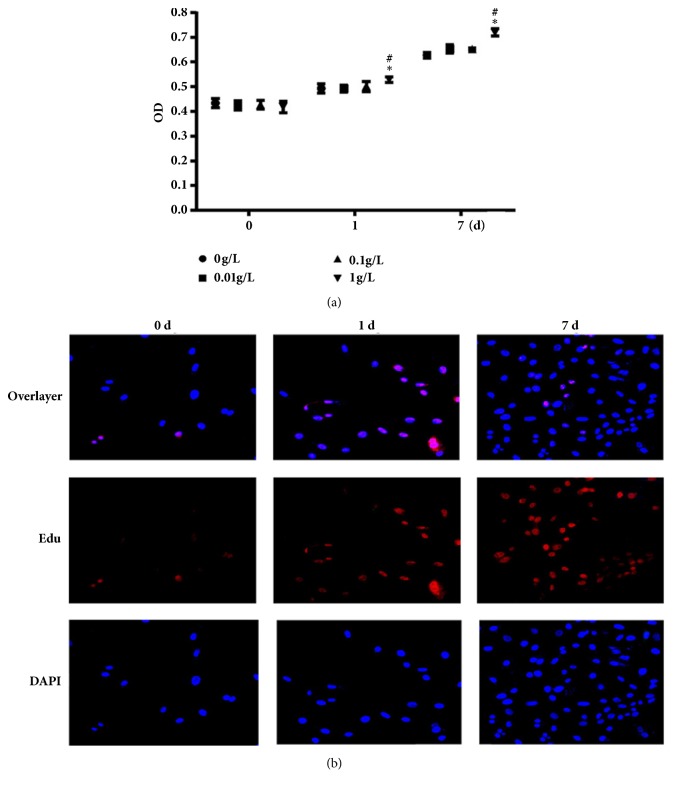
Detection of UHMWPE promote cell proliferation. FLS were incubated with 0-1.0 g/L UHMWPE for 7 d. Cell viability was determined by MTT assay, n=5 (a). FLS were incubated with 1.0 g/L UHMWPE for 0, 1, and 7 d. The images of Edu staining was observed by fluorescence microscope (400x, final magnification), n=5 (b). Cell nuclei were visualized by DAPI staining in blue and proliferative cells were visualized by Edu staining in red. Data was obtained from three independent experiments; results were shown as means ± standard deviation. ^*∗*^p < 0.05, compared with dose of 0 g/L; ^#^p < 0.05, compared with 0 d.

The human primary fibroblast-like synovial (FLS) cells treated UHMWPE for different time and different dose. The proliferation of FLS cells was detected by MTT and Edu staining assay, respectively. As shown in Figure 2(a), UHMWPE could increase the proliferation of FLS cells in dose- and time-dependent manners. Edu staining was performed to analyze the rate of DNA synthesis. The results also showed that 1 g/L UHMWPE promoted the cell proliferation obviously, consisting with MTT results (Figure 2(b)).

### 3.3. The Effects of UHMWPE on Apoptosis of FLS Cells

**Figure 3:**
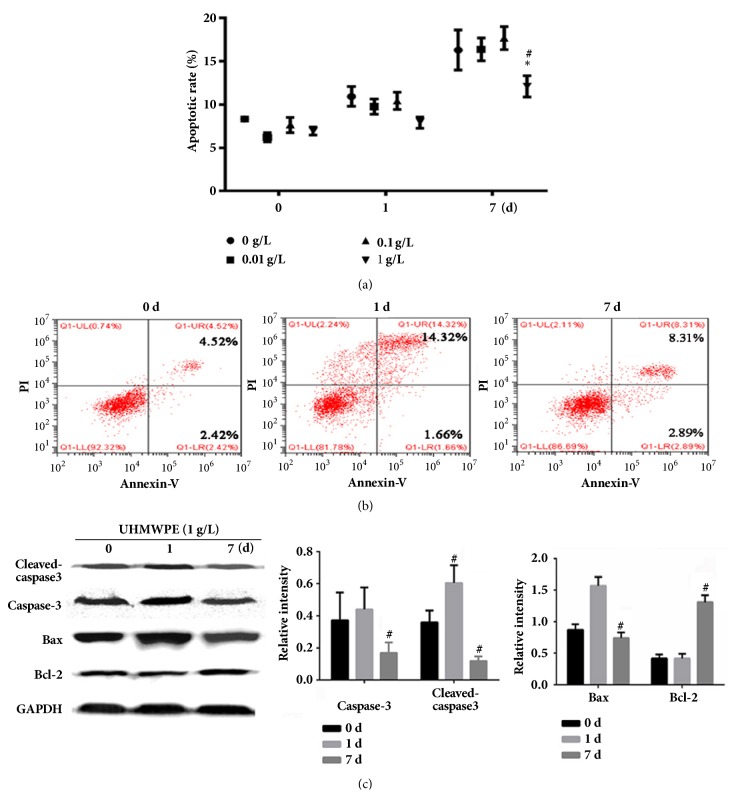
Effects of UHMWPE on apoptosis of FLS cells. FLS were incubated with 0-1.0 g/L UHMWPE for 0, 1, and 7 d or incubated with 1.0 g/L UHMWPE for 0, 1, and 7 d. Apoptotic and necrotic cell populations were analyzed by flow cytometry; quantitative analysis of apoptotic cells after UHMWPE treatment, n=5 (a and b). The expression of apoptosis-related proteins in FLS cells was detected by Western blot assay and the changes of Bcl-2, Bax, caspase-3, and cleaved-caspase-3 were statistically analyzed, n=5 (c). Data was obtained from three independent experiments; results were shown as means ± standard deviation. ^*∗*^p < 0.05, compared with dose of 0 g/L; ^#^p < 0.05, compared with 0 d.

Cells in the lower right quadrant (Q1-LU) represent early apoptotic cells, and cells in the upper right quadrant (Q1-LR) represent late apoptotic and necrotic cells. As shown in Figure 3(a), UHMWPE could increase the apoptosis of FLS cells in time-dependent manner, but 0 ~ 1 g/L UHMWPE incubated for 7 d, the apoptotic rate was decreased. For the FLS cells treated with 1 g/L UHMWPE for 0, 1, and 7 d, the apoptotic rate (Q1-LU+Q1-LU) was 6.94%, 15.98%, and 11.2%, respectively (Figure 3(b)). Next, we analyzed several key proteins involved in apoptosis. Western blots indicated that UHMWPE could upregulate the level of the antiapoptotic protein Bcl-2 and downregulate the levels of proapoptotic proteins Bax, caspase-3, and cleaved-caspase-3 (Figure 3(c)).

### 3.4. UHMWPE Promotes the Secretion of Inflammatory Factor in FLS

**Figure 4:**
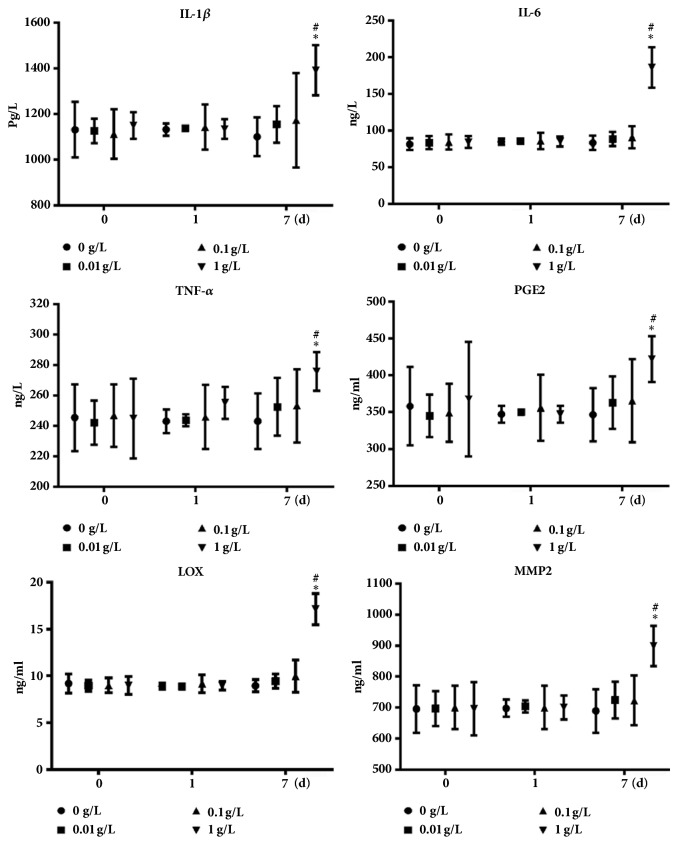
UHMWPE increased the expression of inflammatory factors. The inflammatory factors (IL-6, IL-1*β*, TNF-*α*, and PGE2) and invasion cytokines (MMP2 and LOX) were detected by ELISA, n=5. ^*∗*^p < 0.05, compared with dose of 0 g/L; ^#^p < 0.05, compared with 0 d. Data was obtained from three independent experiments; results were shown as means ± standard deviation.

The FLS cells inflammatory response to UHMWPE was detected by ELISA. After treating 1g/L UHMWPE for 7 d in FLS cells, the levels of IL-6, IL-1*β*, TNF-*α*, PGE2 lipoxidase (LOX), and matrix metalloproteinase-2 (MMP2) all increased significantly (Figure 4).

### 3.5. UHMWPE Promotes the Expression of mIL-6R Protein in FLS Cells

**Figure 5:**
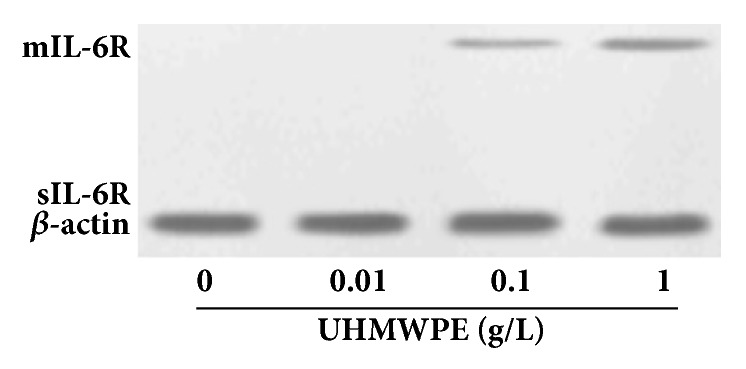
**UHMWPE increased the expression of mIL-6R protein. **FLS cells were incubated with UHMWPE (0, 0.01, 0.1, and 1g/L) for 7 d. The expression of IL-6R protein was detected by western blotting assay.

There are mainly two forms of interleukin-6 receptor (IL-6R), including membrane bound IL-6R (mIL-6R) and soluble IL-6R (sIL-6R), with molecular weights of 80kd and 50kd, respectively [9]. As shown in Figure 5, the expression level of mIL-6R protein was markedly increased after treated with 0.1 and 1g/L UHMWPE for 7 d, while the sIL-6R protein was not detected in FLS cells. These results showed that the UHMWPE could induce the expression of mIL-6R protein in FLS cells.

### 3.6. IL-6RA Combined with UHMWPE Weaken Antiapoptosis Effect in FLS Cells

**Figure 6:**
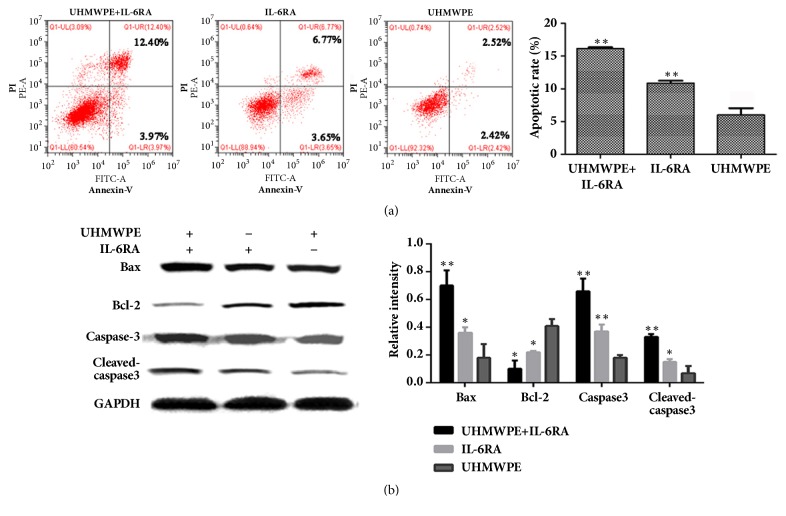
The antagonistic effect of IL-6RA on FLS cells treated with UHMWPE for 7 d. The experiment was divided into 3 groups, including UHMWPE (1 g/L), UHMWPE (1 g/L) + IL-6RA (50 *μ*g/ml), and IL-6RA(50 *μ*g/ml). Apoptotic rate was detected by flow cytometry after treatment IL-6RA, n=5 (a). The expression levels of apoptosis-related proteins including Bax, Bcl2, caspase-3, and cleaved-caspase-3 were analyzed by Western blot after treatment IL-6RA, n=5 (b). Data was obtained from three independent experiments; results were shown as means ± standard deviation. ^*∗*^p < 0.05; ^*∗∗*^p < 0.01 compared with UHMWPE group.

To determine the correlation between apoptosis and cytokines. We examined the apoptotic rate and apoptosis-related proteins in FLS cells in the presence or absence of Interleukin-6 receptor antagonist (IL-6RA). Flow cytometry results showed that the apoptotic rate of FLS cells separately was 16.15%, 10.85%, and 6.03%, which increased significantly after treatment IL-6RA for 7 d (Figure 6(a)). Western blot results showed that the proteins of Bax, caspase-3, and cleaved-caspase-3 all were substantially increased after treatment IL-6RA for 7 d, and the antiapoptotic protein Bcl-2 was decreased significantly (Figure 6(b)).

## 4. Discussion

Aseptic loosening is an important reason for joint replacement after artificial joint replacement. In the metal-polyethylene friction interface, a large number of UHMWPE was produced [10]. The pathologic inflammation induced by UHMWPE may be responsible for aseptic loosening. Macrophages in the membrane tissues swallowed the UHMWPE particles and produced a large number of inflammatory cytokines [11, 12]. The cytokines stimulate the aggregation and activation of osteoclasts, and osteolysis took place and finally leaded to aseptic loosening. The pathological damage was complex and involved many cells, including macrophages, osteoblasts, synovial cells, macrophages which were the focus [13, 14]. The abnormal hyperplasia and inflammation secretion of synovial cells in rheumatoid arthritis have recently been noticed [15, 16]. In this study, we chose the FLS cells as the cell model and UHMWPE as the test substance to observe the effect of UHMWPE on cell activity, inflammatory factor secretion, and apoptosis. Results showed that FLS cells were actively proliferated and grew up quickly, the cell viability was increased significantly compared with the control group after treatment UHMWPE which indicated that UHMWPE not only had no toxicity to FLS cells, but also stimulated the proliferation of cells through some noncontact activation mechanisms [17].

Apoptosis is the process of initiative cell suicide controlled by genes, which is essential for the removal of otherize and maintaining the homeostasis of the cells [18]. Numerous studies have shown that apoptosis plays an important role in aseptic loosening. Landgraeber et al. confirmed that the existence of apoptosis of macrophages, foreign body giant cells, and T lymphocyte in the membrane tissue of artificial joint and with the expression of apoptosis gene include caspase-3, Bax, and p53 [19]. Also, the apoptosis induced by UHMWPE was also involved in the pathological process of aseptic loosening. Petit et al. reported that UHMWPE could induce apoptosis of macrophages to participate in the process of aseptic loosening by inducing TNF-*α* and promoting cascade reaction of caspase-3 [20]. Our results showed that UHMWPE could upregulate the antiapoptosis protein Bcl-2 and downregulate the proapoptosis proteins Bax, caspase-3, and cleaved-caspase-3 in FLS cells which indicated that UHMWPE could negatively regulate the proliferation of FLS cells.

The synovial cells of implant surrounding tissues swallowed particles will secreted inflammatory mediator included TNF-a, IL-1, IL-6, NO, etc., which induced the differentiation and maturation of osteoclasts and caused adjacent bone resorption and bone reconstruction disorders [21]. In our study, the inflammatory factors of IL-6, IL-1*β*, TNF-*α*, PGE2, MMP2, and LOX were significantly increased in FLS cells after treatment UHMWPE; it had the same trend with cell proliferation.

We have demonstrated that the inflammation and apoptosis were all involved in the proliferation of FLS cells after treatment UHMWPE. But the connection between inflammation and apoptosis has not been reported. Previous study has reported that IL-6 could resist cell apoptosis, while IL-6 receptor antagonists (IL-6RA) could antagonize the effect [22, 23]. The IL-6RA exerts its biological activity via inhibition of binding between IL-6 and IL-6R [24]. However, the FLS cells do not generally express IL-6R; thus, we speculated that the IL-6R will be expressed in FLS cells after treatment with UHMWPE. In our study, the level of mIL-6R protein was significantly increased in FLS cells after incubated with 0.1 and 1g/L UHMWPE for 7 d. We further assessed the effects of IL-6RA. Interestingly, we found that the IL-6RA reversed the antiapoptosis effect of UHMWPE on FLS cells though regulating the expression levels of apoptosis-related genes, consistent with other people's reports [25].

Taken together, we provided the first evidence that UHMWPE promote proliferation in FLS cells via antiapoptosis, and the inflammatory factors play an antagonistic apoptosis role. Thus, reversal effect of IL-6RA on late anti-inflammation was important for prosthesis implant.

## Data Availability

The data used to support the findings of this study are available from the corresponding author upon request.
